# HTLV-1 Tax and HBZ cooperatively promote leukemogenesis through miR-155-mediated PTEN suppression and PI3K-Akt activation

**DOI:** 10.1128/jvi.00554-26

**Published:** 2026-06-04

**Authors:** Xiaoru Xin, Yu Mao, Qianan Li, Xinyi Wang, Jinyong Fang, Tiejun Zhao

**Affiliations:** 1 College of Life Sciences, Zhejiang Normal University 66344, Jinhua, China; 2 Department of Hematology, Affiliated Jinhua Hospital, Zhejiang University School of Medicine, Jinhua, China; 3 School of Medicine, Hangzhou City University 728194, Hangzhou, China; Icahn School of Medicine at Mount Sinai, New York, New York, USA

**Keywords:** HTLV-1, ATLL, miR-155, HBZ, Tax, PTEN

## Abstract

Human T-cell leukemia virus type 1 (HTLV-1) causes adult T-cell leukemia/lymphoma (ATLL), but the full scope of its oncogenic mechanisms remains elusive. While the viral oncoprotein Tax drives early oncogenesis, its expression is frequently silenced in later stages of ATLL, whereas the HTLV-1 basic leucine zipper factor (HBZ) is constitutively expressed throughout infection. This shifting viral expression profile underscores the critical need to define HBZ-specific oncogenic mechanisms. Here, we identify miR-155 as a key oncogenic driver in ATLL and reveal a novel, HBZ-dependent post-transcriptional regulatory axis that sustains its overexpression. We confirm Tax-mediated transcriptional activation of miR-155 but further demonstrate that HBZ enhances miR-155 maturation by elevating Dicer expression and promoting its processing of pre-miR-155. The elevated miR-155 promotes tumorigenesis through targeting PTEN and activating PI3K-Akt pathway. Functional studies demonstrate that miR-155 overexpression drives ATLL progression by enhancing proliferation and inhibiting apoptosis, while its inhibition reverses these malignant phenotypes. Xenograft models confirm that miR-155 blockade significantly reduces tumor growth. Our results uncover a cooperative mechanism by which HTLV-1 Tax and HBZ jointly drive miR-155 overexpression to promote leukemogenesis and identify miR-155 as a potential therapeutic target in ATLL.

**IMPORTANCE:**

Adult T-cell leukemia/lymphoma (ATLL) is an aggressive cancer caused by HTLV-1 with limited treatment options. We show that HTLV-1 hijacks the host microRNA miR-155 to drive tumor growth. While the viral protein Tax activates miR-155 transcription, we discovered that HBZ—constitutively expressed even when Tax is silenced—sustains miR-155 expression by upregulating Dicer and enhancing miR-155 processing. Elevated miR-155 then suppresses PTEN and activates the PI3K-Akt pathway, promoting cancer cell proliferation. Importantly, blocking miR-155 significantly reduces tumor growth in mouse models, identifying miR-155 as a promising therapeutic target for ATLL.

## INTRODUCTION

Human T-cell leukemia virus type 1 (HTLV-1), the first discovered human retrovirus, encodes not only the structural genes *Gag*, *Env*, and *Pol* but also several regulatory/accessory proteins, including Tax, Rex, p30, p13, p12, and HBZ. Although CD4^+^ T cells are the principal targets for HTLV-1 infection and transformation, the vast majority of infections remain asymptomatic. However, approximately 5% of carriers eventually develop adult T-cell leukemia/lymphoma (ATLL), a highly aggressive malignancy with challenging diagnosis and poor prognosis (1, 2). Current treatment strategies, including multi-agent chemotherapy, zidovudine/interferon-α (AZT/IFNα), and hematopoietic stem cell transplantation, remain largely non-specific (3), offering limited improvement in long-term survival. Thus, a deeper understanding of the molecular mechanisms underlying HTLV-1-induced oncogenesis is urgently needed.

The insidious pathogenesis of HTLV-1 has significantly hindered the development of effective prevention and treatment strategies for ATLL (4). Among viral factors, the Tax and HBZ proteins play central roles in leukemogenesis (5). Tax acts as the primary initiator of cellular transformation, whereas HBZ is critical for sustaining the proliferative capacity of transformed cells. Notably, HBZ is constitutively expressed in ATLL cells and remains functionally active even during late-stage disease when Tax expression is commonly silenced (6). Beyond viral proteins, accumulating evidence indicates that host genetic and epigenetic alterations are also indispensable for HTLV-1-induced malignancy (7, 8). A landmark genomic and transcriptomic analysis of 426 ATLL patients revealed widespread mutational and epigenetic dysregulation (9), reinforcing their essential contribution to ATLL pathogenesis. Nevertheless, the precise mechanisms by which epigenetic remodeling influences HTLV-1-mediated transformation remain poorly understood.

MicroRNAs (miRNAs), a class of short non-coding RNAs, are key epigenetic regulators that modulate gene expression by binding to the 3′ untranslated region (3′UTR) of target mRNAs, thereby influencing diverse physiological and pathological processes. Numerous studies have established strong connections between miRNAs and viral infections. Some viruses encode their own viral miRNAs (v-miRNAs) to facilitate persistence and pathogenicity. For instance, bovine leukemia virus (BLV)-derived miRNAs promote viral replication by modulating host genes (10). Other viruses, though lacking miRNA-coding capacity, exploit host miRNAs to their advantage, as observed with avian leukosis virus subgroup J (ALV-J) (11), human immunodeficiency virus (HIV) (12), and hepatitis B virus (HBV) (13). Similarly, HTLV-1, which does not encode miRNAs, hijacks host miRNA networks. Infection-induced dysregulation of cellular miRNAs is increasingly recognized as a critical driver to ATLL development (14, 15).

Among these, miR-155 is a well-characterized multifunctional miRNA involved in hematopoiesis, immune responses, and various human cancers. Its dysregulation is frequently associated with viral pathogenesis. For example, elevated miR-155 in HIV progressors suppresses innate immune responses and accelerates disease progression (16). In the context of HTLV-1, Bellon et al. reported as early as 2009 that miR-155 was upregulated in HTLV-1-infected cells (17). Then, study reported that interferon regulatory factor 4 (IRF4) could promote the transcription of the *BIC* gene and thereby increase the expression of miR-155 in the samples of ATLL patients (18). Subsequently, Tomita et al. demonstrated that Tax could trans-activate miR-155 via NF-κB and AP-1 pathways and that its overexpression promoted the growth of HTLV-1-transformed cells (19). Notably, the miR-155 inhibitor cobomarsen (MRG-106) has been shown to suppress proliferation and induce apoptosis in HTLV-1-positive cutaneous T-cell lymphoma (CTCL) cells *in vitro* (20). An early-phase clinical trial evaluating cobomarsen (NCT02580552) in several lymphomas, including CTCL and ATLL, has been initiated, highlighting the translational interest in targeting miR-155. These findings strongly suggest that miR-155 plays a critical role in HTLV-1-induced leukemogenesis, yet its precise mechanistic contributions and therapeutic relevance in ATLL remain incompletely elucidated. Especially, given that Tax expression is frequently silenced in later stages of ATLL, whereas HBZ remains constitutively expressed (6); the precise mechanisms sustaining miR-155 overexpression, particularly those independent of Tax, are yet poorly defined.

In this study, we aim to elucidate the mechanisms underlying sustained miR-155 overexpression in ATLL, with a particular focus on the role of the constitutively expressed HBZ protein beyond the established Tax-dependent pathway. We identify HTLV-1-induced miR-155 upregulation as a critical oncogenic driver in ATLL. While confirming Tax-dependent transcriptional activation, we uncover a novel mechanism whereby HBZ independently enhances miR-155 expression via post-transcriptional regulation. Functionally, elevated miR-155 promotes ATLL progression by activating the PI3K-Akt signaling through targeted suppression of PTEN. Using both *in vitro* and *in vivo* models, we demonstrate that miR-155 overexpression accelerates tumorigenesis, whereas its inhibition suppresses malignant phenotypes and impairs xenograft growth. Our findings not only establish miR-155 as a promising therapeutic target in ATLL but also reveal a previously unrecognized role for HBZ in modulating miRNA-dependent oncogenic signaling.

## MATERIALS AND METHODS

### Cell culture

HTLV-1 positive T-cell lines (Hut-102, MT-1, MT-2, MT-4, ATL-2, ATL-T, TL-Om1), HTLV-1 negative human T-cell lines (Jurkat, Hut-78, Molt-4, Cemt-4), Jurkat cell lines stably expressing the spliced form of HBZ (Jurkat-HBZ) and control (Jurkat-ctrl), and JPX-9 cells were cultured in RPMI 1640 medium with 10% fetal bovine serum (FBS), L-glutamine, and antibiotics. 293T cells were cultured in Dulbecco’s modified Eagle’s medium (DMEM) with 10% FBS, L-glutamine, and antibiotics.

### Plasmids, lentivirus, and reagents

Expression plasmids for pX1MT-M, Myc-his-HBZ, Flag-HBZ, and phRL-TK were maintained in our laboratory stock. The pCK-V5-Dicer plasmid was kindly provided by V Narry Kim (21) of Seoul National University. The miR-155 promoter report plasmid was kindly provided by Erik K. Flemington (22) of Tulane University. The wild-type and mutant PTEN 3′-UTR luciferase reporters were constructed to contain either the native miR-155 binding sequence (5′-GCAUUAA-3′) or a mutated version (5′-CCGTAAT-3′) designed to disrupt miR-155 binding. Wild-type and mutant PTEN 3′-UTR reporter plasmids were generated by Sangon Biotech. Expression plasmids for pCMV-miR-155 and pCMV6-XL5-PTEN were purchased from Origene. Lentivirus rLV-miR and rLV-miR-155 were constructed by Wu Han Viral Therapy Technologies. The inhibitor for miR-155 and NC were purchased from Thermo Fisher Scientific. The shDicer, siTax, and siHBZ were purchased from Sangon Biotech. CdCl_2_ was purchased from Millipore.

### Quantitative real-time PCR

Total RNA was isolated from cells cultured *in vitro* or tissues of xenografted tumors with TRIzol reagent (Thermo Fisher Scientific), and RNA concentration was measured spectrophotometrically. Reverse transcription was carried out using the FastKing RT Kit (TIANGEN) for general genes and the miRcute Plus miRNA First-Strand cDNA Kit (TIANGEN) for miRNA. Real-time qPCR was performed using the PowerUp SYBR Green Master Mix (Thermo Fisher Scientific) for general cDNA and the miRcute Plus miRNA qPCR Kit for miRNA. Primers used in this study were shown in Table S1. For mRNA quantification, target cDNA amplification was normalized to 18S ribosomal RNA (rRNA) expression. For miRNA quantification, expression levels were normalized to U6 small nuclear RNA (snRNA). Relative expression of target mRNA/miRNA were calculated using the 2^−△△Ct^ method.

### Luciferase reporter assay

Jurkat cells were seeded on 12-well plates at 1.0 × 10^5^ cells per well. After 24 h, cells were transfected with the firefly luciferase reporter plasmid (containing the promoter or 3′-UTR of interest), the indicated expression plasmids, and the Renilla luciferase control plasmid (phRL-TK) using Lipofectamine LTX with Plus Reagent (Thermo Fisher Scientific). After 48 h, the luciferase assay was carried out using the Dual-Luciferase Reporter Assay System (Promega). Firefly luciferase activity (reporter) was normalized to Renilla luciferase activity (transfection control). Data represent mean ± SD of three independent experiments performed in triplicate.

### Western blot

The 293T cells were transfected with indicated plasmids using Lipofectamine 2000 Reagent (Thermo Fisher Scientific). After 48 h, cells were collected and Western blotting were performed as previously described (23). For the endogenous protein, cell lysates were collected and incubated with indicated antibodies, and the following protocol was same as in 293T cells. Antibodies used in this study were as follows: Anti-Myc and anti-Flag were purchased from Sigma. Anti-Tax, anti-Dicer, anti-PTEN, anti-PI3K, anti-AKT, and anti-p-AKT were purchased from Santa Cruz Biotechnology. Anti-GAPDH was from Sangon Biotech.

### RNA immunoprecipitation

293T cells were co-transfected with the expression plasmids of pCMV-pre-miR-155, V5-Dicer, and Flag-HBZ using Lipofectamine 2000. Forty-eight hours after transfection, cells were harvested for RNA immunoprecipitation. Cell lysates were prepared and incubated with 1 μg of anti-Dicer antibody or control IgG overnight at 4°C with gentle rotation. The pretreated protein A/G agarose beads (Smart-Lifesciences) were added to the antibody-lysate mixture and incubated for 4 h at 4°C. The bead-antibody-RNA complexes were obtained by centrifugation at 3,400 rpm for 3 min at 4°C and washed five times with ice-cold wash buffer. RNA was extracted from the immunoprecipitates using TRIzol reagent following standard procedures. The relative enrichment of pre-miR-155 in the immunoprecipitates was quantified by real-time PCR.

### Co-immunoprecipitation assay

293T cells were co-transfected with Flag-HBZ and V5-Dicer. After 48 h, cells were lysed and incubated with anti-Flag antibody overnight at 4°C. Protein A/G agarose beads were added to capture immune complexes. After washing, bound proteins were eluted and analyzed by Western blot using anti-Flag and anti-Dicer antibodies.

### Co-culture assay

To assess the effects of cell-contact-mediated HTLV-1 infection on miR-155 expression, a co-culture system was employed. Prior to co-culture, HTLV-1-positive ATL-T cells were treated with Mitomycin C (MMC; 100 μg/mL) for 1 h to inhibit their proliferation. Subsequently, the pretreated ATL-T cells were co-cultured with HTLV-1-negative Jurkat cells at ratios of 1:1, 1:2, and 1:3, respectively. Under these conditions, MMC-treated ATL-T cells remained viable for initial viral transmission but underwent substantial cell death (>95%) by 48 h, allowing for the specific analysis of the surviving Jurkat population. After 48 h of co-culture, Jurkat cells were harvested, and the expression levels of pre-miR-155 and mature miR-155 were analyzed by real-time PCR.

### CCK-8 assay

Jurkat-rLV, Jurkat-rLV-miR-155, ATL-T, and ATL-2 cells were seeded in 96-well plates. Cell viability was assessed at 0-, 24-, and 48-h time points by adding 10 μL of CCK-8 reagent (MeilunBio) to each well followed by incubation at 37°C for 3 h. Absorbance was measured at 450 nm using a microplate reader, and the growth curve was generated using GraphPad Prism software.

### Colony formation assay

ATL-T cells were seeded at a density of 300 cells per well in 6-well plates and allowed to adhere overnight. The cultures were then maintained in complete growth medium at 37°C with 5% CO₂ for 12–14 days. After cell cloning, the medium was discarded, 1.7 mL methanol was added for 10 min, and 1 mL crystal violet staining solution was added for 20 min. The colonies were photographed and counted.

### Apoptosis analysis

#### Flow cytometry for cell apoptosis

Jurkat-rLV and Jurkat-rLV-miR-155 cells were seeded in 24-well plates and treated with 5 μM camptothecin for 5 h to induce apoptosis. After treatment, cells were harvested, washed twice with cold PBS, and resuspended at a density of 1 × 10⁵ cells per tube in 100 μL of 1 × binding buffer. For apoptosis detection, cells were gently mixed with 5 μL of Annexin V-FITC and 5 μL of propidium iodide (PI) (20 μg/mL), followed by incubation in the dark at room temperature for 15 min. To ensure uniform staining, the tubes were gently tapped every 5 min during incubation. Apoptotic cells were immediately quantified using flow cytometry.

#### TUNEL assay for tissue apoptosis

Apoptotic cells in paraffin-embedded tumor tissue sections harvested from the xenograft mouse model were detected using the TUNEL (TdT-mediated dUTP Nick-End Labeling) method. Following deparaffinization and rehydration, tissue sections were permeabilized with proteinase K (20 μg/mL) for 20 min at 37°C. The sections were then incubated with TUNEL reaction mixture containing TdT enzyme and red-fluorescent-dye-labeled dUTP for 1 h at 37°C in a humidified dark chamber. After washing, cell nuclei were counterstained with DAPI (4′,6-diamidino-2-phenylindole) for 10 min at room temperature. The stained sections were mounted with anti-fade mounting medium and visualized under a fluorescence microscope. Apoptotic cells were identified by red fluorescent labeling, while all cell nuclei appeared blue under DAPI fluorescence.

### Wound healing assay

ATL-T cells were seeded in 6-well plates and transfected with either the miR-155 inhibitor or NC using Lipofectamine 2000. After 24 h of transfection (upon reaching ~90% confluency), a uniform scratch wound was generated in the cell monolayer using a sterile 10-μL pipette tip. Following wound creation, detached cells and debris were removed by gentle PBS washing. To quantify cell migration, three random fields of view (FOVs) per well were imaged immediately after scratching (0 h) and re-examined at 24 h post-scratch. The identical FOVs were tracked to ensure consistent comparison. Migration rates were assessed based on wound closure over time.

### *In vivo* tumorigenicity assay

ATL-T cells were harvested and resuspended in sterile phosphate-buffered saline (PBS). Twelve 6-week-old male NSG mice (Hangzhou Ziyuan Laboratory Animal Technology Co., Ltd.) were subcutaneously injected with 50 μL of 4 × 10^6^ ATL-T cells combined with 50 μL of Matrigel (BDBIO). When tumors became palpable (at day 12 after injection), mice were randomly divided into a control group (injecting 10 nmol/mouse antagomiR NC) and an experimental group (injecting 10 nmol/mouse hsa-miR-155-5p antagomiR). The intra-tumor injections were repeated every three days for a total of four times. The tumor volume was measured with caliper every 3 days using the formula: volume = (length × width^2^)/2. The experiment was terminated at day 27 when all tumors were surgically excised for subsequent molecular analyses including real-time PCR and immunohistochemistry. All animal procedures were conducted in strict compliance with protocols approved by the Institutional Animal Care and Use Committee of Zhejiang Normal University.

### Immunohistochemistry analysis

Tissue sections embedded in paraffin were subjected to standard deparaffinization using xylene and rehydrated through a graded ethanol series. Antigen retrieval was performed by heating the sections in citrate buffer (pH 6.0) at 95°C for 20 min. Endogenous peroxidase activity was quenched by treatment with 3% hydrogen peroxide for 15 min at room temperature. The sections were then incubated overnight at 4°C with the primary antibody against Ki67. After washing, the sections were incubated with biotinylated secondary antibody for 1 h at room temperature, followed by streptavidin-peroxidase conjugate for 30 min. Immunoreactivity was visualized using 3,3′-diaminobenzidine (DAB) as the chromogen, with hematoxylin counterstaining to highlight cellular morphology. Stained sections were evaluated by light microscopy, with images captured at appropriate magnifications.

### Statistical analysis

All experiments were performed at least three times. Data are presented as mean ± SD. For comparisons involving more than two groups, one-way ANOVA with Tukey’s multiple comparison test was used. For two-group comparisons, unpaired two-tailed Student’s *t*-test was applied. For the xenograft tumor growth experiment, tumor volumes over time were analyzed using two-way ANOVA with repeated measures, followed by Bonferroni’s post-hoc test for comparisons at individual time points. Tumor weights at endpoint were analyzed using unpaired two-tailed Student’s *t*-test. Significance levels were defined as *P < 0.05, **P < 0.01, ***P < 0.001 and ns, not significant.

## RESULTS

### miRNA profiles in ATLL patients and characterization of miR-155 expression in HTLV-1 positive T-cell lines

**Fig 1:**
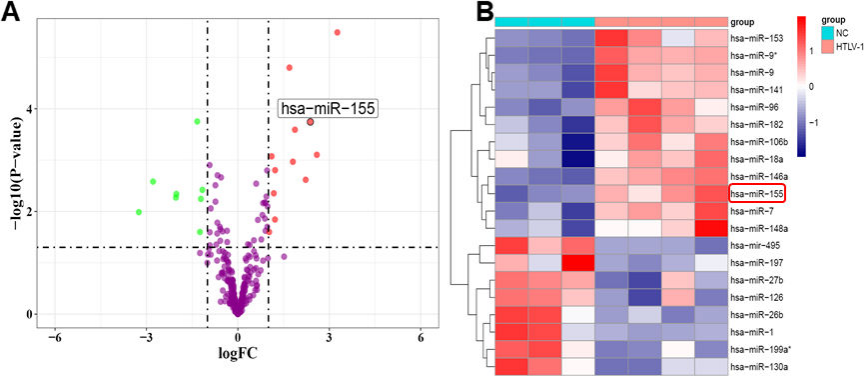
miRNA expression profiling in ATLL patients. (**A**) Volcano plot showing differentially expressed miRNAs in peripheral blood mononuclear cells from ATLL patients (*n* = 4) compared to healthy donors (*n* = 3). Data were analyzed from the GEO data set GSE11577. Red dots represent significantly upregulated miRNAs (LogFC ≥ 1, *P* < 0.05), and green dots represent significantly downregulated miRNAs (LogFC ≤ −1, *P* < 0.05). miR-155 is labeled. (**B**) Unsupervised hierarchical clustering heatmap of the 20 significantly dysregulated miRNAs (12 upregulated and 8 downregulated) from the same data set, demonstrating distinct miRNA expression patterns between ATLL patients and controls.

To characterize miRNA expression profiling in ATLL patients and HTLV-1 transformed cell lines, we first analyzed the publicly available microarray data set GSE11577 (24) from the GEO database. This data set included samples from four ATLL patients and three healthy controls, and thus the findings should be considered preliminary. Using a threshold of ∣LogFC∣≥ 1 and *P* < 0.05, we identified 12 upregulated and 8 downregulated miRNAs (Fig. 1A and B). Among these, miR-155 emerged as one of the most significantly upregulated miRNAs in this data set (Table S2). This observation served as a hypothesis-generating starting point for subsequent experimental validation in HTLV-1-positive cell lines and functional studies.

**Fig 2:**
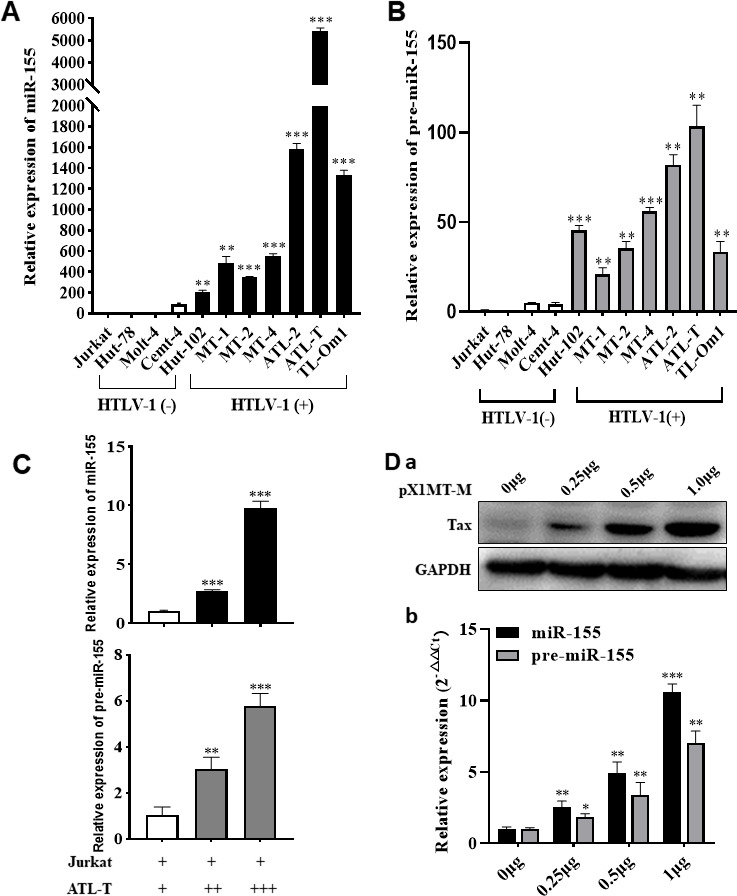
HTLV-1 infection induces the expression of mature miR-155 and its precursor. (**A**) Relative expression levels of mature miR-155 in a panel of HTLV-1-positive T-cell lines (Hut-102, MT-1, MT-2, MT-4, ATL-2, ATL-T, TL-Om1) compared to HTLV-1-negative T-cell lines (Jurkat, Hut-78, Molt-4, Cemt-4), as determined by qRT-PCR. Data are normalized to 18s rRNA. (**B**) Relative expression levels of pre-miR-155 in the same cell lines as in panel A. (**C**) Jurkat cells were mock-treated or co-cultured with increasing ratios of irradiated HTLV-1-producing ATL-T cells to allow viral infection. After 48 hours, the expression of mature miR-155 and pre-miR-155 in Jurkat cells was measured by qRT-PCR. (**D**) 293T cells were transfected with increasing amounts of the HTLV-1 infectious molecular clone pX1MT-M. The expression of mature miR-155 and pre-miR-155 was assessed by qRT-PCR at 48 hours post-transfection. Data in all panels are presented as mean ± SD from at least three independent experiments. For comparisons involving more than two groups (**C and D**), one-way ANOVA with Tukey’s multiple comparison test was used. For two-group comparisons, unpaired two-tailed Student’s *t*-test was applied. *P < 0.05, **P < 0.01, ***P < 0.001.

We next validated miR-155 expression in seven HTLV-1 positive T-cell lines (Hut-102, MT-1, MT-2, MT-4, ATL-2, ATL-T, TL-Om1) compared to four HTLV-1 negative T-cell lines (Jurkat, Hut-78, Molt-4, Cemt-4). Consistent with the patient data, miR-155 was markedly elevated in all HTLV-1 positive lines (Fig. 2A). Quantitative real-time PCR further revealed upregulation of its precursor, pre-miR-155, in the same set of HTLV-1-positive cells (Fig. 2B). To directly assess the correlation between HTLV-1 infection and miR-155 induction, we established a coculture system in which Jurkat cells were cocultured with HTLV-1-producing ATL-T cells. After 48 h, both mature miR-155 and pre-miR-155 levels were significantly elevated in Jurkat cells, and their expression levels progressively increased with higher numbers of ATL-T cells used (Fig. 2C). Notably, mature miR-155 expression exceeded that of its precursor under all conditions tested. We further confirmed these findings by transfecting 293T cells with the infectious HTLV-1 molecular clone pX1MT-M. Again, both miR-155 and pre-miR-155 were upregulated, showing a gradual increase with higher transfection doses, with mature miR-155 showing more pronounced induction than its precursor (Fig. 2D).

Collectively, these results demonstrate that HTLV-1 infection drives elevated expression of miR-155 in both clinical ATLL samples and experimental infection models.

### HTLV-1 upregulates miR-155 expression through the coordinated actions of Tax and HBZ

Given the established roles of Tax and HBZ as key viral oncoproteins in HTLV-1 pathogenesis (25–27), we investigated their potential involvement in miR-155 upregulation. We first assessed the role of Tax using the JPX-9 cell line, in which Tax expression is inducible by CdCl₂. Western blot analysis confirmed successful Tax induction (Fig. S1A). Subsequent qRT-PCR analysis revealed that both mature miR-155 and pre-miR-155 progressively increased upon Tax induction. Notably, pre-miR-155 exhibited a more pronounced increase than the mature form (Fig. S1B). Consistent with previous findings (19), a luciferase reporter assay demonstrated that Tax transactivates the miR-155 promoter in an NF-κB-dependent manner (Fig. S1C and D). These results indicate that Tax enhances miR-155 expression at the transcriptional level.

**Fig 3:**
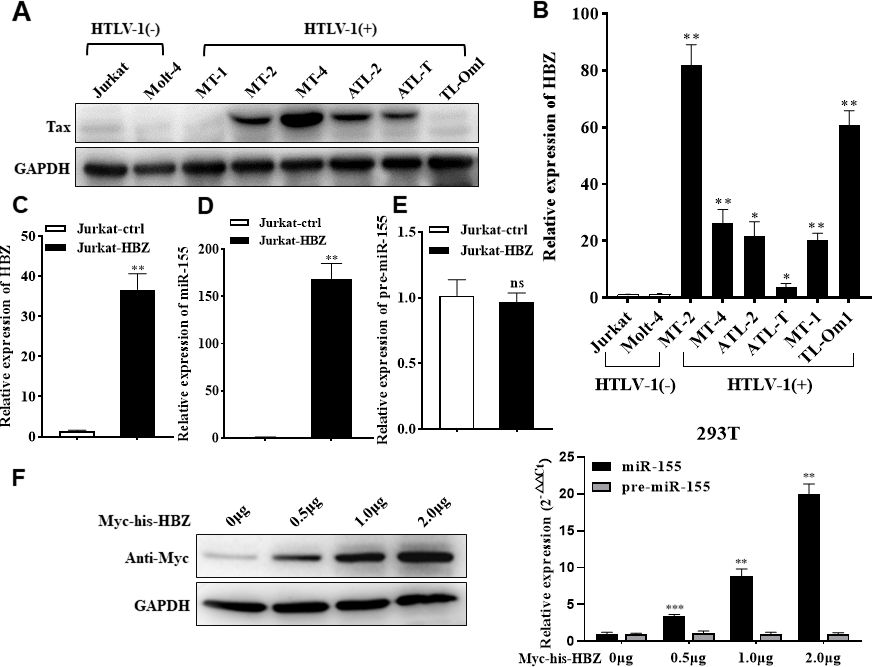
The HTLV-1 protein HBZ upregulates mature miR-155 expression post-transcriptionally. (**A**) Western blot analysis of Tax protein expression in a panel of six HTLV-1-positive T-cell lines. GAPDH serves as a loading control. (**B**) Quantitative RT-PCR analysis of HBZ mRNA expression in the same panel of HTLV-1-positive T-cell lines as in panel A. Data are normalized to 18s rRNA. (**C**) Quantitative RT-PCR confirming the stable expression of the spliced isoform of HBZ in Jurkat-HBZ cells compared to Jurkat-Ctrl cells. Data are normalized to 18s rRNA. (**D and E**) Quantitative RT-PCR analysis of mature miR-155 (**D**) and pre-miR-155 (**E**) expression in Jurkat-HBZ and Jurkat-Ctrl cells. Data are normalized to 18s rRNA. (**F**) (left) 293T cells were transfected with increasing amounts of a Myc-His-HBZ expression plasmid. Western blot shows dose-dependent HBZ expression. (right) Quantitative RT-PCR analysis of mature miR-155 and pre-miR-155 in the same samples. Data in panels **B–F** are presented as mean ± SD from three independent experiments. *P < 0.05, **P < 0.01, ***P < 0.001, ns, not significant (Student’s *t*-test).

Since Tax expression is frequently silenced in approximately half of all ATL cases, while HBZ is constitutively expressed in all cases (6), we hypothesized that HBZ may also contribute to miR-155 regulation. We examined the expression of Tax and HBZ in six HTLV-1 positive T-cell lines (MT-1, MT-2, MT-4, ATL-2, ATL-T, TL-Om1). Western blot and qRT-PCR analyses confirmed the absence of Tax protein in MT-1 and TL-Om1 cells (Fig. 3A; Fig. S1E), whereas HBZ was constitutively expressed across all lines (Fig. 3B). The observed upregulation of miR-155 in these Tax-negative lines (as shown in Fig. 2A) suggested an alternative mechanism sustaining miR-155 expression independent of Tax.

To test whether HBZ regulates miR-155, we utilized Jurkat cell lines stably expressing the spliced isoform of HBZ (Jurkat-HBZ). Western blot confirmed high HBZ expression in Jurkat-HBZ cells compared to mock-transfected controls (Fig. 3C). qRT-PCR analysis showed a significant increase in mature miR-155 levels in Jurkat-HBZ cells (Fig. 3D), whereas pre-miR-155 expression remained unchanged (Fig. 3E). This effect was further validated in 293T cells transfected with a Myc-His-HBZ expression plasmid. Mature miR-155 levels increased dose-dependently with HBZ expression (Fig. 3F), while pre-miR-155 levels were again unaffected. To further assess the relationship between Tax and HBZ in regulating miR-155, we knocked down Tax or HBZ alone or together in HTLV-1-infected cells. Knockdown of either Tax or HBZ alone attenuated miR-155 expression, whereas double knockdown showed no additive effect (Fig. S1F), indicating that Tax and HBZ act cooperatively rather than synergistically.

Collectively, these findings demonstrate that HTLV-1 drives miR-155 overexpression through both Tax and HBZ. Tax acts primarily by transcriptionally activating the miR-155 promoter, whereas HBZ upregulates mature miR-155 through a post-transcriptional mechanism that does not involve modulating its precursor.

### HBZ promotes miR-155 maturation via Dicer at the posttranscriptional level

**Fig 4:**
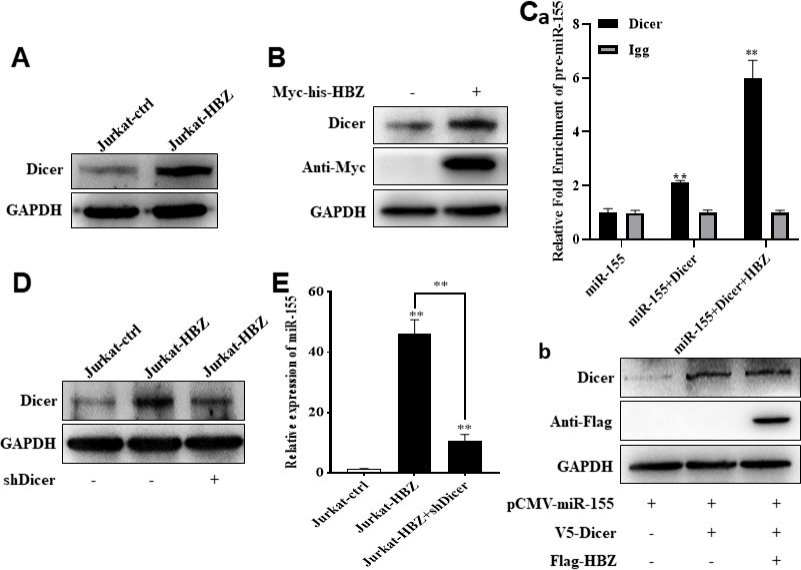
HBZ promotes the maturation of miR-155 by upregulating Dicer and enhancing its binding to pre-miR-155. (**A**) Western blot analysis of Dicer protein expression in Jurkat-HBZ and Jurkat-Ctrl cells. (**B**) Western blot analysis of Dicer and HBZ (Myc-tag) expression in 293T cells transfected with a Myc-His-HBZ plasmid. (**C**) RNA immunoprecipitation (RIP) assay using an anti-Dicer antibody in 293T cells co-transfected with pre-miR-155, Dicer, and HBZ expression plasmids. The amount of co-precipitated pre-miR-155 was quantified by qRT-PCR and expressed as a percentage of the input RNA. (**D**) Western blot confirming the knockdown efficiency of Dicer in Jurkat-HBZ cells transduced with Dicer-specific shRNA (shDicer). (**E**) Quantitative RT-PCR analysis of mature miR-155 expression in Jurkat-HBZ cells from the experiment in panel D. Data are normalized to 18s rRNA. Data in panels C and E are presented as mean ± SD from three independent experiments. *P < 0.05, **P < 0.01, ***P < 0.001 (Student’s *t*-test).

To clarify the mechanism by which HBZ promotes the expression of miR-155, we first performed a luciferase reporter assay. As shown in Fig. S2A, HBZ did not significantly affect miR-155 promoter activity, consistent with its lack of effect on pre-miR-155 levels (Fig. 3E and F). Notably, although HBZ alone had no effect on miR-155 promoter activity, it suppressed Tax-induced NF-κB-dependent activation of the promoter (Fig. S2B), suggesting a complex interplay between Tax and HBZ in regulating miR-155 transcription. Next, we studied whether HBZ modulates the biogenesis process of miR-155 at the posttranscriptional level. We analyzed the expression of key genes involved in miRNA biogenesis using qRT-PCR. Among the candidates—including *Drosha*, *Dgcr8*, *Exportin-5*, *Dicer1*, *Ago2,* and *Ago3*—only Dicer1 mRNA was significantly upregulated by HBZ (Fig. S2C). Western blot analysis further confirmed that Dicer protein levels were elevated in both Jurkat-HBZ cells (Fig. 4A) and in 293T cells transfected with HBZ (Fig. 4B).

Since Dicer is essential for cleaving pre-miRNAs into mature miRNAs (28, 29), we hypothesized that HBZ enhances Dicer-mediated processing of pre-miR-155. Using RNA immunoprecipitation (RIP) in 293T cells co-expressing pre-miR-155, Dicer, and HBZ, we observed that HBZ significantly increased the binding of Dicer to pre-miR-155 (Fig. 4C). To determine whether HBZ directly interacts with Dicer, we performed co-immunoprecipitation (Co-IP) assays. The results showed that HBZ does not physically interact with Dicer (Fig. S2D), suggesting that the enhanced binding between Dicer and pre-miR-155 is likely due to the increased Dicer protein levels upon HBZ expression rather than a direct physical interaction. To functionally validate this mechanism, we knocked down Dicer expression in Jurkat-HBZ cells using shRNA (Fig. 4D). Dicer knockdown markedly attenuated the HBZ-induced upregulation of mature miR-155 (Fig. 4E), confirming that Dicer is required for HBZ-mediated miR-155 maturation.

In summary, these findings demonstrate that HBZ enhances miR-155 expression post-transcriptionally by upregulating Dicer and facilitating its binding to and processing of pre-miR-155.

### miR-155 activates the PI3K-Akt signaling pathway by targeting PTEN in a HTLV-1-dependent manner

**Fig 5:**
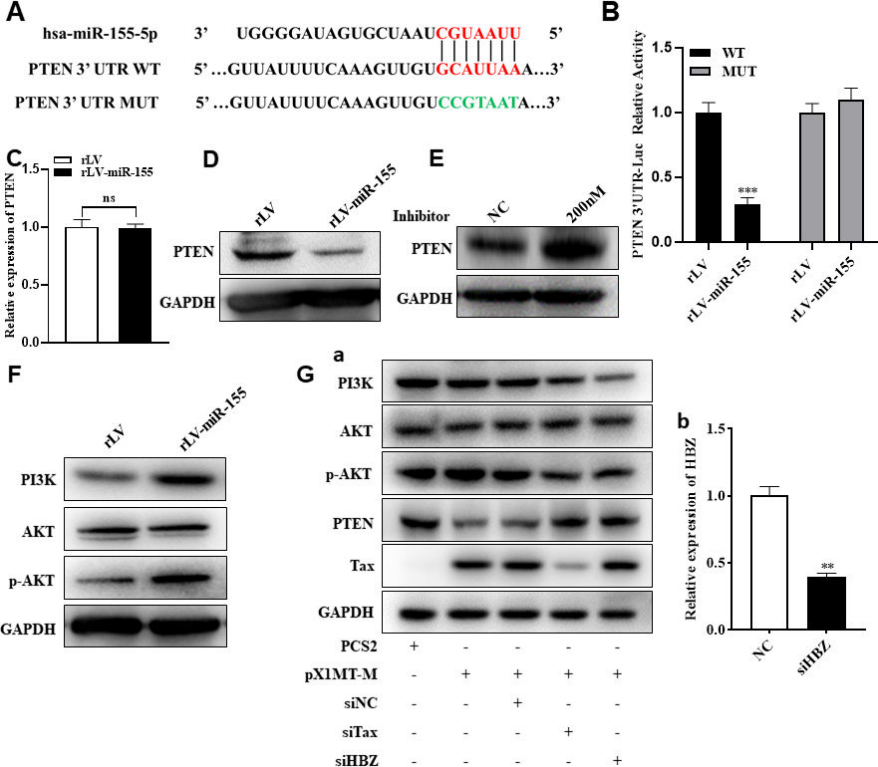
miR-155 activates the PI3K-Akt signaling pathway by targeting PTEN in a HTLV-1-dependent manner. (**A**) Schematic representation of the predicted miR-155-5p binding site in the wild-type (WT) PTEN 3′UTR and the mutant (MUT) construct used for luciferase assays. (**B**) Luciferase reporter activity in Jurkat cells stably overexpressing miR-155 (rLV-miR-155) or control (rLV) after transfection with PTEN 3′UTR-WT or MUT reporter constructs. (**C**) PTEN mRNA levels in rLV-miR-155 and rLV control cells measured by qRT-PCR. (**D**) Western blot analysis of PTEN protein expression in rLV-miR-155 and rLV control cells. (**E**) Western blot analysis of PTEN expression in ATL-T cells transfected with miR-155 inhibitor or negative control (NC). (**F**) Western blot analysis of PI3K, phosphorylated Akt (p-Akt), and total Akt in rLV-miR-155 and rLV control cells. (**G**) (**a**) Western blot analysis of Tax, PTEN, PI3K, p-Akt, and total Akt in 293T cells co-transfected with pX1MT-T and siRNAs targeting Tax (siTax) or HBZ (siHBZ). Knockdown of either viral protein attenuated miR-155-mediated PTEN suppression and PI3K-Akt pathway activation. (**b**) HBZ mRNA levels measured by qRT-PCR. Data in panels B, C, and G are presented as mean ± SD from three independent experiments. **P < 0.01, ***P < 0.001, ns, not significant (Student’s *t*-test).

To explore the functional role of miR-155 in ATLL, we used miRTargetLink 2.0 (https://ccb-compute.cs.uni-saarland.de/mirtargetlink2) to identify its potential targets. Among the candidates, PTEN was selected due to the presence of a highly conserved binding site for miR-155-5p within its 3′-UTR (Fig. 5A). To validate this interaction, we constructed a lentivirus overexpressing the miR-155 precursor (rLV-ZsGreen-miR-155) and established stable Jurkat cell lines. Overexpression of both pre-miR-155 and mature miR-155 was confirmed by qRT-PCR (Fig. S3A and B). We then cloned either the wild-type (WT) or a mutant (MUT) PTEN 3′-UTR into a luciferase reporter vector (Fig. 5A). Luciferase assays revealed that miR-155 overexpression significantly suppressed the activity of the WT 3′-UTR, but not the MUT construct (Fig. 5B), confirming direct targeting via the predicted seed region. Consistent with this, miR-155 overexpression had no effect on PTEN mRNA levels (Fig. 5C) but substantially reduced PTEN protein expression (Fig. 5D). Conversely, inhibition of miR-155 in ATL-T and ATL-2 cells increased PTEN protein levels (Fig. 5E; Fig. S3C). Given PTEN’s role as a key negative regulator of the PI3K-Akt pathway, we assessed pathway activation. Overexpression of miR-155 led to increased levels of PI3K and phosphorylated Akt (p-Akt) (Fig. 5F), indicating enhanced pathway activity. Finally, to determine whether HTLV-1 regulates this axis, we knocked down Tax or HBZ in 293T cells transfected with the HTLV-1 molecular clone pX1MT-T. Depletion of either viral protein (Fig. 5G) reduced miR-155-mediated suppression of PTEN and subsequent activation of PI3K-Akt signaling (Fig. 5H).

Together, these results demonstrate that HTLV-1-induced miR-155 directly targets PTEN, leading to activation of the PI3K-Akt pathway, and that this oncogenic signaling axis depends on the expression of Tax and HBZ.

### miR-155 accelerates ATLL cell proliferation by targeting PTEN *in vitro*

**Fig 6:**
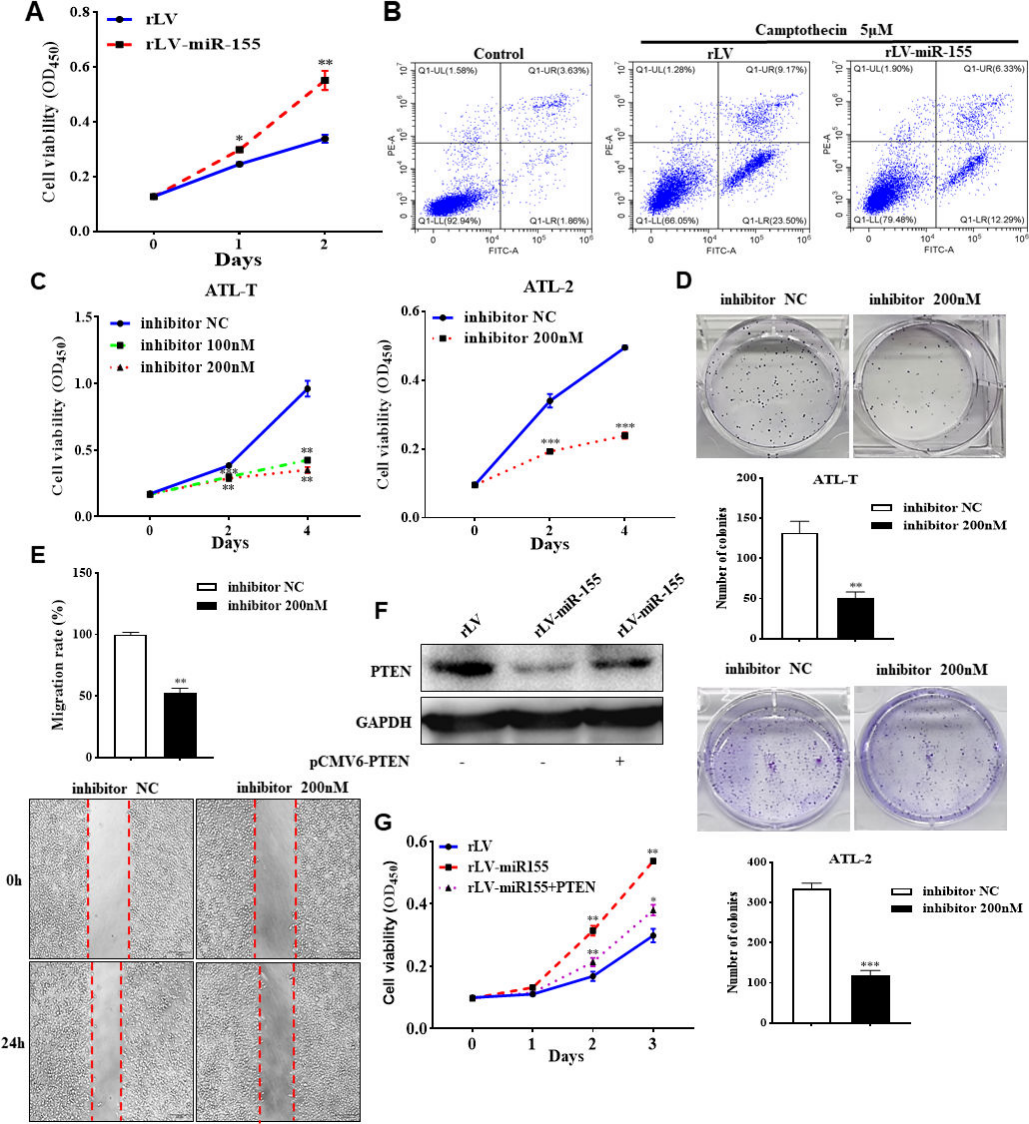
miR-155 promotes proliferation and inhibits apoptosis of ATLL cells by targeting PTEN *in vitro*. (**A**) Cell proliferation measured by CCK-8 assay in Jurkat cells stably overexpressing miR-155 (rLV-miR-155) or control (rLV). (**B**) Apoptosis analysis by flow cytometry in Jurkat cells from panel A after induction of apoptosis with camptothecin. (**C**) Cell proliferation measured by CCK-8 assay in ATL-T and ATL-2 cells transfected with miR-155 inhibitor or negative control (NC). (**D**) Colony formation assays in ATL-T and ATL-2 cells transfected with miR-155 inhibitor or NC. The lower panel shows quantitative analysis of colony numbers. (**E**) Wound healing assay in ATL-T cells transfected with miR-155 inhibitor or NC. The upper panel shows quantitative analysis of migration rate. (**F**) Western blot analysis of PTEN expression in rLV-miR-155 Jurkat cells transfected with PTEN expression vector (pCMV6-PTEN). (**G**) Cell proliferation measured by CCK-8 assay in rLV-miR-155 Jurkat cells from the experiment in panel F. Data in panels A, C, D, E, and G are presented as mean ± SD from three independent experiments. *P < 0.05, **P < 0.01, ***P < 0.001 (Student’s *t*-test).

To assess the functional significance of miR-155 upregulation in ATLL, we investigated its role in tumorigenesis and cell growth *in vitro*. CCK-8 assays revealed that miR-155 overexpression significantly enhanced the proliferation of Jurkat cells (Fig. 6A). Flow cytometry analysis further demonstrated that miR-155 overexpression markedly suppressed apoptosis (Fig. 6B). Conversely, inhibition of miR-155 significantly impaired the proliferation of ATLL-derived cell lines (ATL-T and ATL-2) (Fig. 6C). Colony formation assays showed that miR-155 depletion strongly reduced the clonogenic capacity of ATL-T and ATL-2 cells (Fig. 6D). In addition, wound-healing assays indicated that miR-155 inhibition significantly attenuated the migratory ability of ATL-T cells (Fig. 6E). Together, these gain- and loss-of-function studies establish that miR-155 promotes the initiation and progression of ATLL *in vitro*.

To determine whether PTEN mediates the proliferative effects of miR-155, we reintroduced PTEN into miR-155-overexpressing Jurkat cells (rLV-miR-155) by plasmid transfection (Fig. 6F). Restoration of PTEN expression significantly attenuated the pro-proliferative effect of miR-155 (Fig. 6G), confirming that miR-155 promotes ATLL cell growth primarily through suppression of PTEN.

### miR-155 promotes ATLL cell proliferation *in vivo*

**Fig 7:**
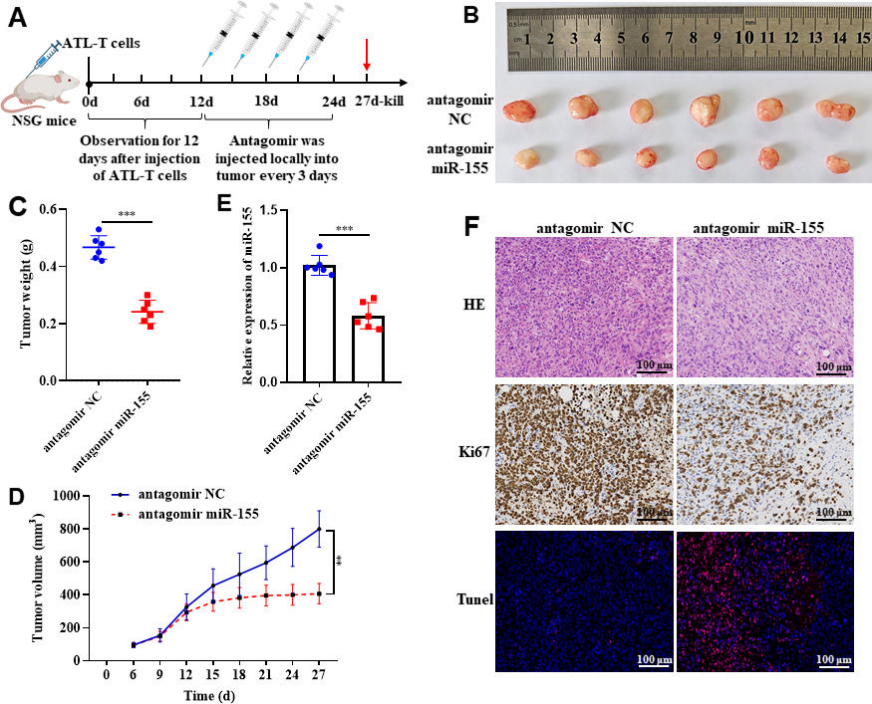
Inhibition of miR-155 suppresses tumor growth in ATLL xenograft models. (**A**) Schematic diagram of the experimental timeline. NSG mice were subcutaneously injected with ATL-T cells (4 × 10⁶). When tumors became palpable (day 12), mice were randomly divided into two groups (*n* = 6) and received intratumoral injections of miR-155 antagomiR or negative control antagomiR (antagomiR NC) every three days for four total injections. Mice were sacrificed on day 27. (**B**) The images of excised tumors from each group at the endpoint. (**C**) Tumor weights measured after dissection at the experimental endpoint. (**D**) Tumor volume measurements recorded every three days during the treatment period. (**E**) qRT-PCR analysis of miR-155 expression in xenograft tumor tissues, normalized to 18s rRNA. (**F**) (Top) H&E staining of tumor sections. (Middle) Immunohistochemical staining for Ki67. (Bottom) TUNEL staining for apoptosis detection. Scale bar: 100 μm. Data in panels C, D, and E are presented as mean ± SD (*n* = 6). *P < 0.05, **P < 0.01, ***P < 0.001 (Student’s *t*-test).

To further evaluate the oncogenic role of miR-155 *in vivo*, we established ATL-T cell xenografts in NSG mice. When tumors became palpable (day 12), mice were randomly divided into two groups (*n* = 6 per group) and received intratumoral injections of either miR-155 antagomiR or negative control antagomiR (antagomiR NC) every 3 days for a total of four injections. Tumors were harvested on day 27 (Fig. 7A), as outlined in the experimental timeline (Fig. 7A). Excised tumors are shown in Fig. 7B. Consistent with our *in vitro* findings, inhibition of miR-155 significantly impaired the tumorigenic capacity of ATL-T cells, as evidenced by reduced tumor weight and volume (Fig. 7C and D). qRT-PCR analysis confirmed successful suppression of miR-155 in the miR-155 antagomir-treated group (Fig. 7E). Histological analysis by H&E staining revealed a marked decrease in tumor malignancy upon miR-155 inhibition. Immunohistochemistry further showed reduced Ki67 expression in the miR-155 antagomir-treated group, indicating suppressed tumor cell proliferation. In addition, TUNEL staining demonstrated a significant increase in apoptosis in tumors from mice treated with miR-155 antagomiR (Fig. 7F).

## DISCUSSION

Adult T-cell leukemia/lymphoma (ATLL) is a lymphoproliferative malignancy caused by human T-cell leukemia virus type 1 (HTLV-1). Currently, approximately 20 million people worldwide are infected with HTLV-1, with 3%–5% of carriers developing aggressive ATLL decades after initial infection (30, 31). Given the limited efficacy of current treatments, elucidating the molecular mechanisms underlying HTLV-1-induced leukemogenesis remains imperative. Accumulating evidence suggests that dysregulation of epigenetic modulators, particularly miRNAs, plays a pivotal role in HTLV-1-mediated oncogenesis. For instance, Fochi et al. demonstrated that miR-130b promotes proliferation and survival of HTLV-1-infected cells by suppressing TP53INP1 expression (32). Similarly, Tomita et al. reported that Tax-mediated upregulation of miR-146a enhances proliferation of HTLV-1-infected cells (33).

miR-155, a well-characterized oncomiR, is encoded by the host *BIC* gene located on chromosome 21. Processed from the BIC transcript, mature miR-155 regulates multiple oncogenic pathways and has been implicated in various malignancies (e.g., lung [34], liver [35], breast [36], and cervical cancer [37]). However, its precise role in ATLL pathogenesis remains poorly understood. In this study, we demonstrate that HTLV-1 infection robustly upregulates miR-155 expression in both clinical ATLL samples and HTLV-1-positive cell lines, including those transfected with the HTLV-1 infectious clone pX1MT-M.

The viral regulatory proteins Tax and HBZ are established key players in HTLV-1 oncogenesis. Tax, which drives viral transcription through NF-κB activation (38, 39), is expressed intermittently to evade cytotoxic T lymphocyte (CTL) responses while maintaining infectivity (40). In contrast, HBZ is constitutively expressed due to its low immunogenicity and is essential for proliferation of HTLV-1-infected cells (41). Here, we identify a novel, HBZ-driven mechanism that enhances miR-155 maturation at the post-transcriptional level. Specifically, HBZ upregulates Dicer expression and facilitates its binding to pre-miR-155, thereby promoting miR-155 processing. This HBZ-dependent pathway operates independently of Tax activity and provides a mechanistic explanation for the sustained miR-155 overexpression observed in advanced ATLL, where Tax expression is frequently silenced. Notably, a previous study reported that HBZ downregulates Dicer expression and reduces miR-155 levels in certain cellular contexts (42). The discrepancy with our findings may be attributed to differences in experimental systems (e.g., 293T-HBZ stable cells vs Jurkat-HBZ stable cells) and the fact that we focused specifically on post-transcriptional processing rather than steady-state levels. Further studies are needed to reconcile these observations and to understand how HBZ modulates miRNA biogenesis in a context-dependent manner. While our findings establish a functional link between HBZ and Dicer, the precise molecular mechanism by which HBZ upregulates Dicer expression remains to be elucidated. Future studies are warranted to investigate whether HBZ acts through indirect mechanisms, such as recruitment of transcription factors, modulation of chromatin accessibility, or post-transcriptional regulation, and to determine whether HBZ interacts with other viral proteins, such as Rex, which has been reported to suppress Dicer activity (43), to coordinately regulate miRNA biogenesis. Moreover, the precise interplay between Tax and HBZ in regulating miR-155 requires further investigation. Taken together, our data suggest that HTLV-1 employs a coordinated strategy to achieve miR-155 overexpression: Tax drives transcriptional activation via NF-κB, whereas HBZ suppresses this activation but promotes post-transcriptional processing through Dicer upregulation. This dynamic balance highlights the central importance of miR-155 dysregulation in HTLV-1-driven transformation.

Mechanistically, we show that miR-155 exerts its oncogenic effects by directly targeting the 3′UTR of PTEN, leading to activation of the PI3K-Akt pathway. Although miR-155-mediated PTEN suppression has been previously documented in other cancer types, such as hepatocellular carcinoma (44), our study provides the first evidence that this regulatory axis operates in ATLL cells. Especially, this regulatory axis is HTLV-1-dependent, as inhibition of either Tax or HBZ restores PTEN expression and abrogates PI3K-Akt activation. These observations are consistent with previous studies linking PTEN/PI3K-Akt dysregulation to ATLL pathogenesis. For example, Tax has been shown to competitively bind DLG-1, displacing PTEN and thereby activating Akt (45), while NDRG2-mediated PTEN dephosphorylation enhances PI3K-Akt signaling in ATLL cells (46). In particular, our finding underscores HBZ’s non-redundant role in maintaining the oncogenic drive, especially in the absence of Tax.

Functionally, miR-155 overexpression enhanced proliferation and suppressed apoptosis in Jurkat cells, whereas its inhibition attenuated malignant phenotypes (proliferation, clonogenicity, and migration) in ATLL cells. Rescue experiments confirmed PTEN as the key mediator of miR-155-driven oncogenicity. *In vivo*, antagomiR-mediated suppression of miR-155 significantly impaired tumor growth in ATL-T xenograft models, highlighting its therapeutic potential. The early-phase clinical trial of the miR-155 inhibitor cobomarsen (MRG-106, NCT02580552) across several lymphomas, along with its demonstrated preclinical activity in HTLV-1-positive CTCL cells, supports the translational potential of targeting miR-155 (20). Our findings in ATLL models further strengthen this rationale, though further evaluation of long-term safety and efficacy remains necessary.

In summary, our study identifies a cooperative model in which HTLV-1 Tax and HBZ collectively elevate miR-155 expression to promote ATLL leukemogenesis via the PTEN-PI3K-Akt axis. Importantly, HBZ-mediated post-transcriptional enhancement of miR-155 maturation may represent an immune evasion strategy that supports viral persistence and leukemogenic progression. These findings not only deepen our understanding of HTLV-1 pathogenesis by elucidating a critical, Tax-independent function of HBZ, but also provide an evidence for targeting miR-155 as a therapeutic strategy in ATLL.

This study has two limitations that should be acknowledged. First, the initial bioinformatic screening for dysregulated miRNAs was based on a public data set with a relatively small sample size (four ATLL patients vs three healthy controls). While this analysis served its purpose for hypothesis generation, conclusions drawn from it require validation in larger, independent cohorts. Second, our functional experiments primarily relied on established HTLV-1-positive cell lines. Although these models are valuable tools, they may not fully recapitulate the genetic and phenotypic heterogeneity observed in primary ATLL tumors. Future studies employing patient-derived samples or primary cells would be important to confirm the generalizability of our findings.

### Conclusion

**Fig 8:**
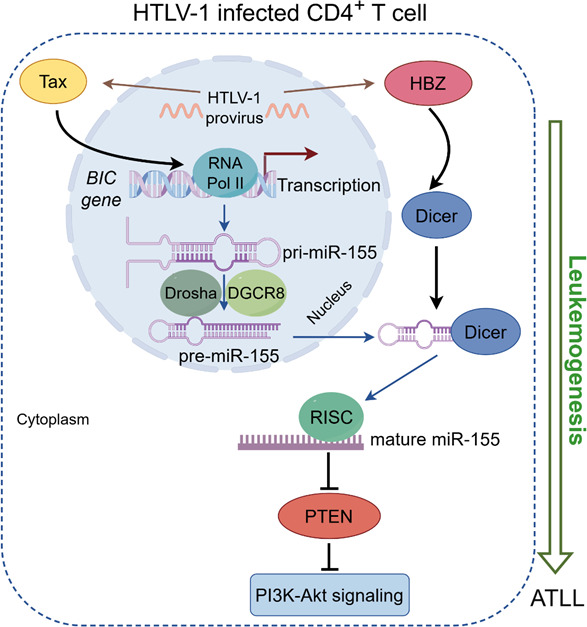
Schematic model illustrating how HTLV-1 promotes ATLL leukemogenesis by activating the miR-155-PTEN-PI3K/Akt signaling axis.

In conclusion, our study establishes a coherent oncogenic axis in which HTLV-1 drives ATLL leukemogenesis through the cooperative upregulation of miR-155 by Tax and HBZ. While Tax activates miR-155 transcription, HBZ post-transcriptionally enhances its maturation via Dicer. The resulting miR-155 overexpression promotes tumorigenesis by directly suppressing PTEN and activating the PI3K-Akt signaling pathway, as validated both *in vitro* and *in vivo*. These findings not only uncover a key Tax-independent function of HBZ in maintaining oncogenic signaling but also highlight miR-155 as a potential therapeutic target in ATLL. A schematic model of this proposed pathway is provided in Fig. 8.

## Data Availability

All data from this study are included in this article and its supplemental material. Uncropped original Western blot images are provided in the supplemental material.

## SUPPLEMENTAL MATERIAL

Figure S1 jvi.00554-26-s0001.TIF The HTLV-1 protein Tax transcriptionally activates miR-155 expression.

Figure S2 jvi.00554-26-s0002.tif HBZ does not activate the miR-155 promoter but transcriptionally upregulates Dicer.

Figure S3 jvi.00554-26-s0003.TIF Validation of miR-155 overexpression and inhibition models.

Raw data jvi.00554-26-s0004.pdf Original western blot images.

Supplemental tables jvi.00554-26-s0005.docx Tables S1 and S2.

ASM does not own the copyrights to Supplemental Material that may be linked to, or accessed through, an article. The authors have granted ASM a non-exclusive, world-wide license to publish the Supplemental Material files. Please contact the corresponding author directly for reuse.
