# Supplementary figures and images for: HTLV-1 Tax and HBZ cooperatively promote leukemogenesis through miR-155-mediated PTEN suppression and PI3K-Akt activation
Source: J Virol. 2026 Jun 4;100(6):e00554-26. doi: 10.1128/jvi.00554-26 (PMC13288991; doi:10.1128/jvi.00554-26)

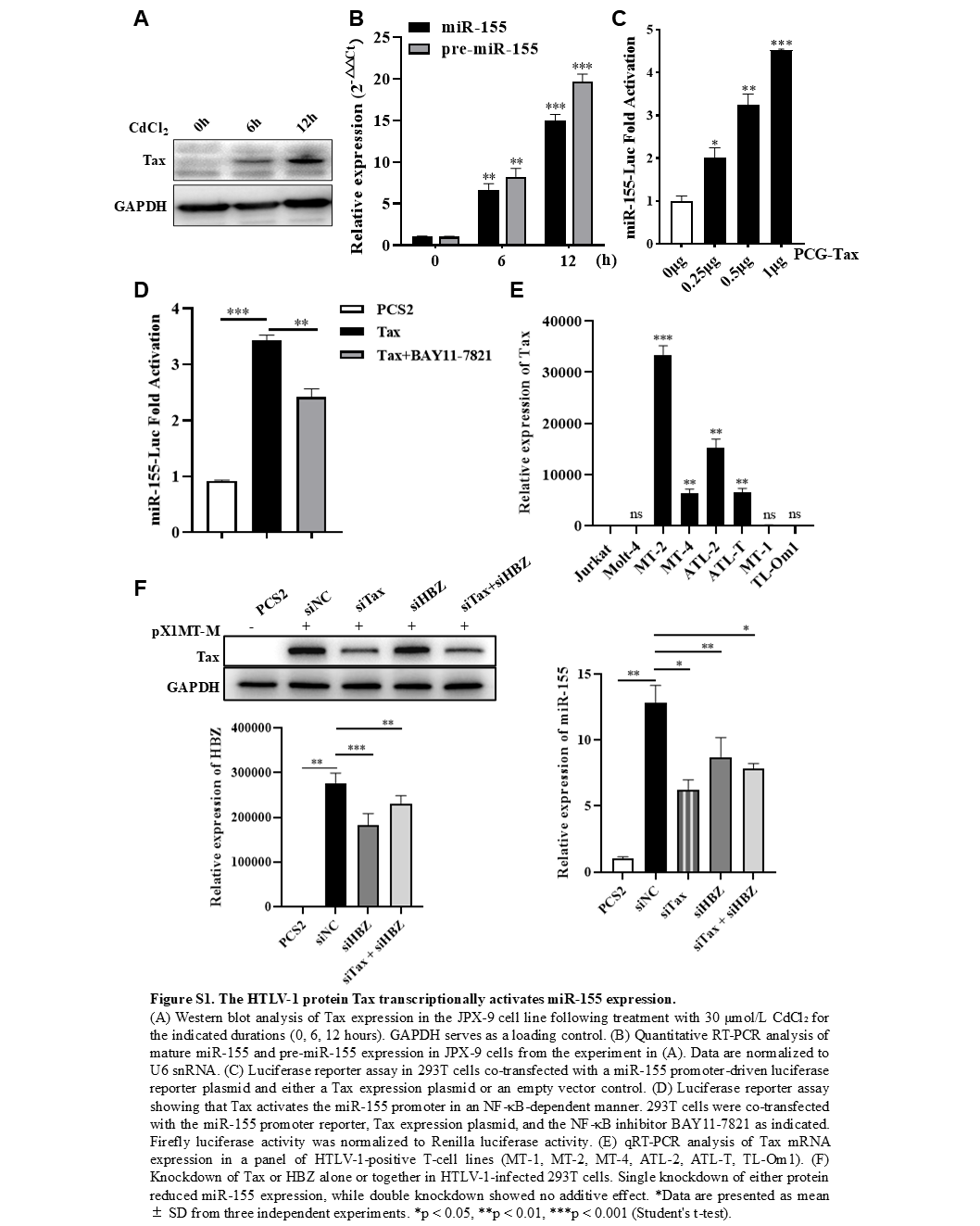

Supplement: Figure S1 — The HTLV-1 protein Tax transcriptionally activates miR-155 expression. [file jvi.00554-26-s0001.tif]

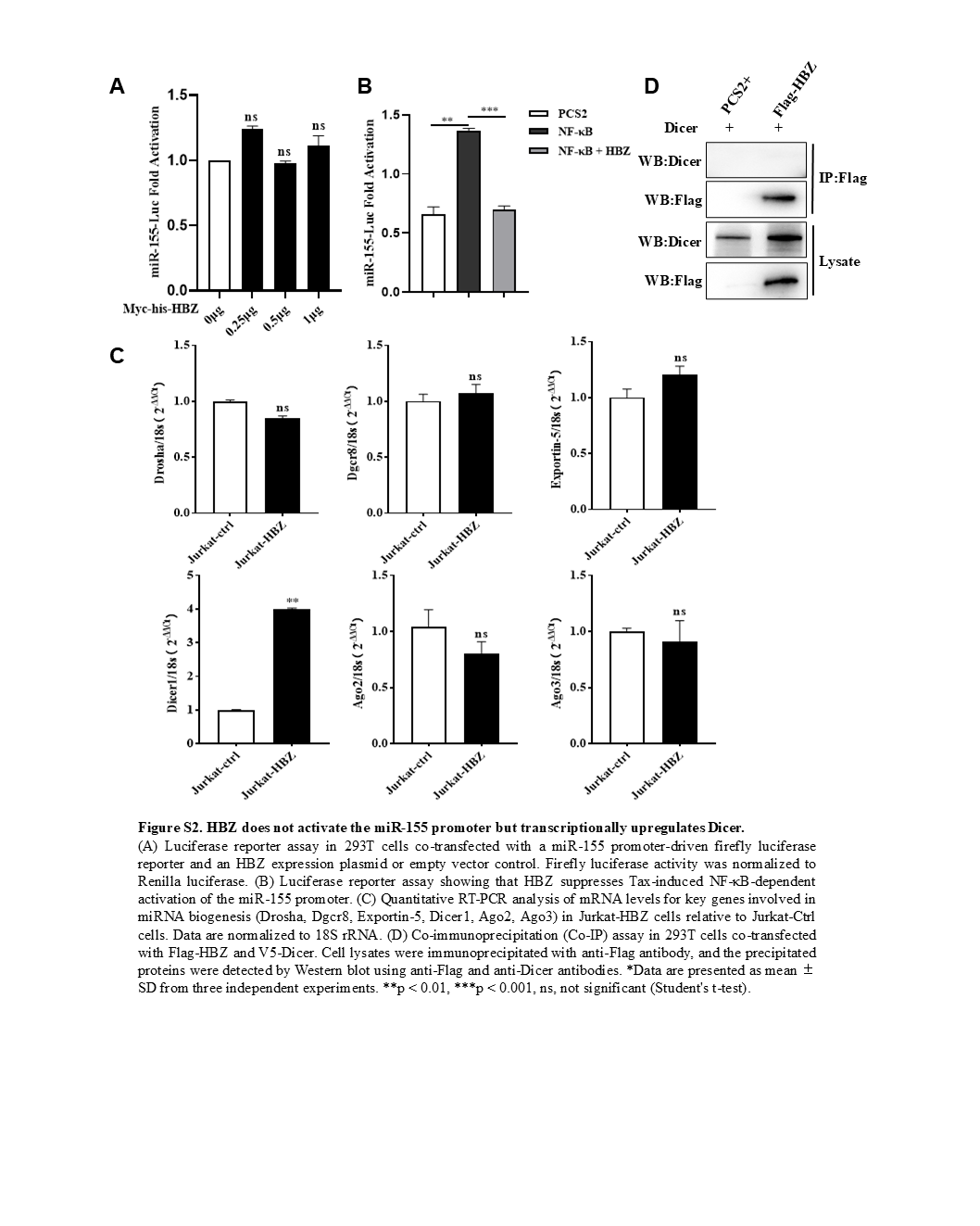

Supplement: Figure S2 — HBZ does not activate the miR-155 promoter but transcriptionally upregulates Dicer. [file jvi.00554-26-s0002.tif]

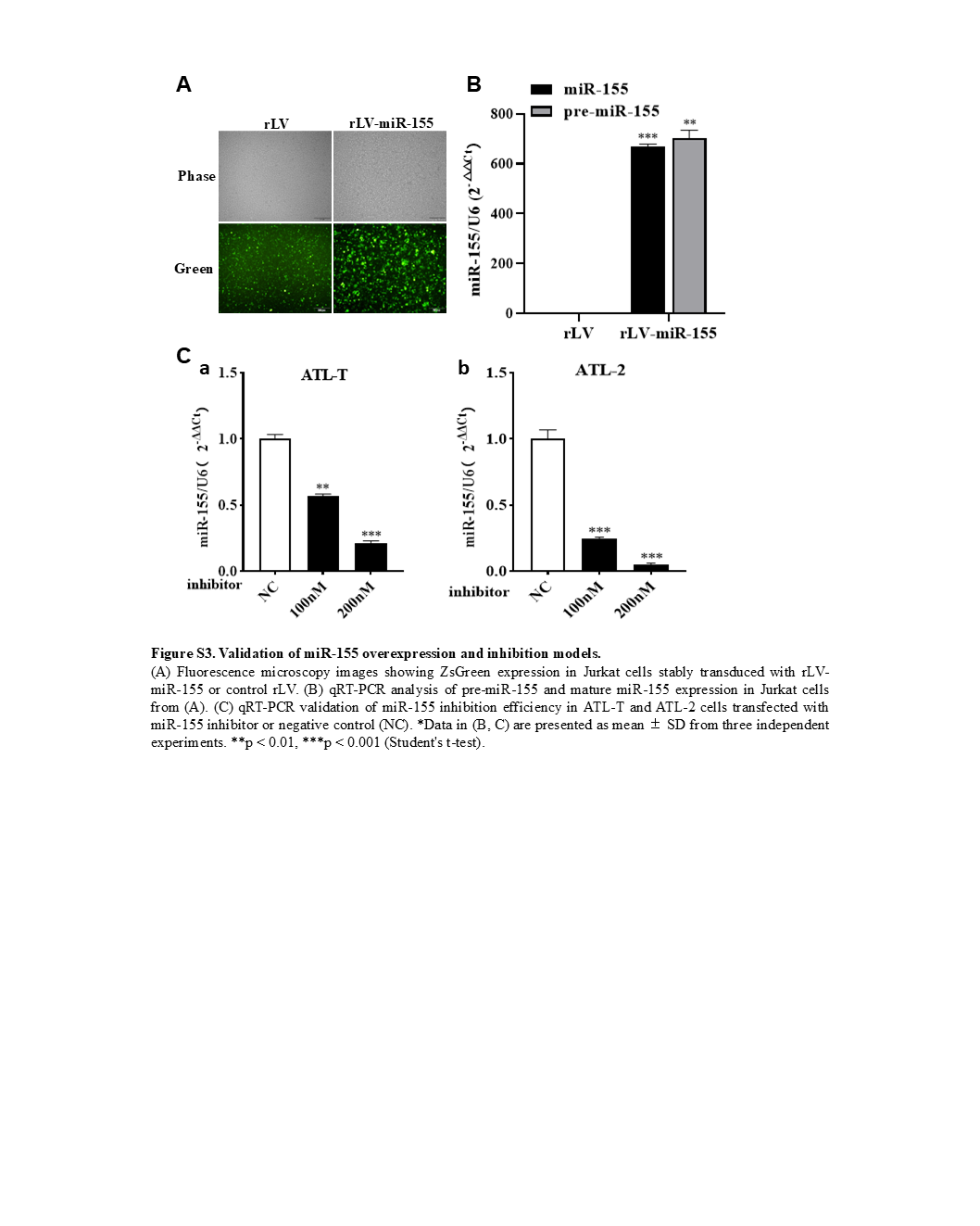

Supplement: Figure S3 — Validation of miR-155 overexpression and inhibition models. [file jvi.00554-26-s0003.tif]
