# Supplementary material for: HTLV-1 Tax and HBZ cooperatively promote leukemogenesis through miR-155-mediated PTEN suppression and PI3K-Akt activation
Source: J Virol. 2026 Jun 4;100(6):e00554-26. doi: 10.1128/jvi.00554-26 (PMC13288991; doi:10.1128/jvi.00554-26)
Supplement: Raw data — Original western blot images. [file jvi.00554-26-s0004.pdf]

Figure 2D 293T cells were transfected with increasing amounts of the HTLV-1 infectious molecular clone pX1MT-M.

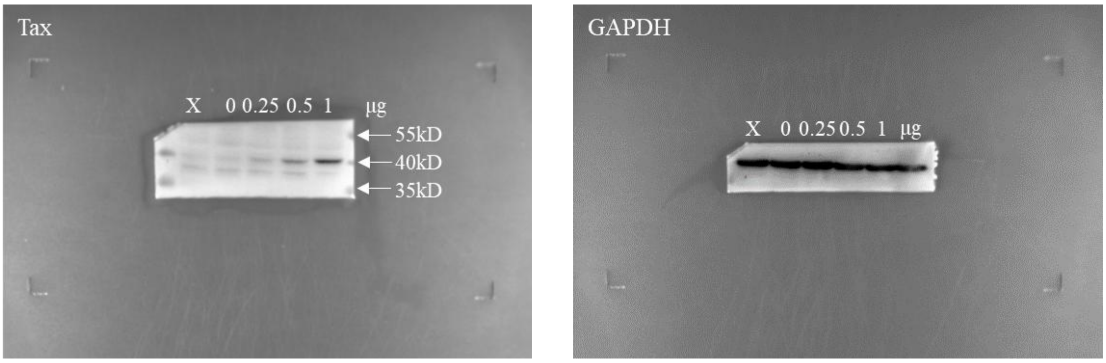

Figure 3A Western blot analysis of Tax protein expression in a panel of six HTLV-1-positive T-cell lines. GAPDH serves as a loading control.

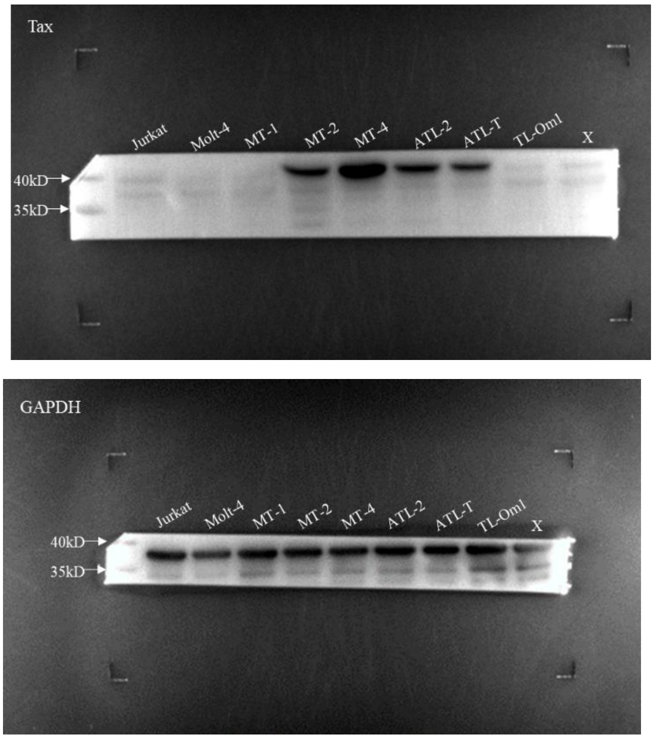

Figure 3F 293T cells were transfected with increasing amounts of a Myc-His-HBZ expression plasmid. Western blot shows dose-dependent HBZ expression.

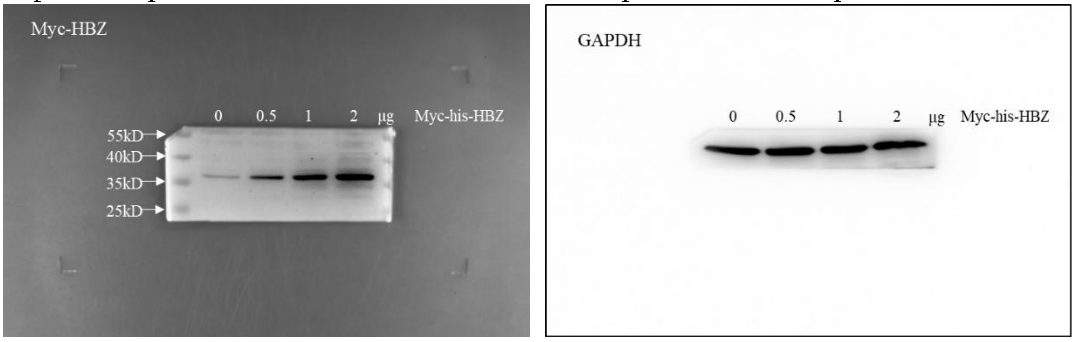

Figure 4A Western blot analysis of Dicer protein expression in Jurkat-HBZ and Jurkat-Ctrl cells.

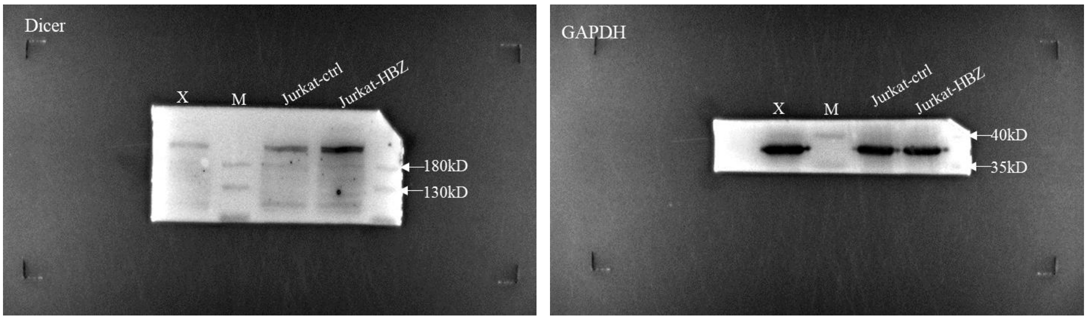

Figure 4C RNA immunoprecipitation (RIP) assay using an anti-Dicer antibody in 293T cells co-transfected with pre-miR-155, Dicer, and HBZ expression plasmids.

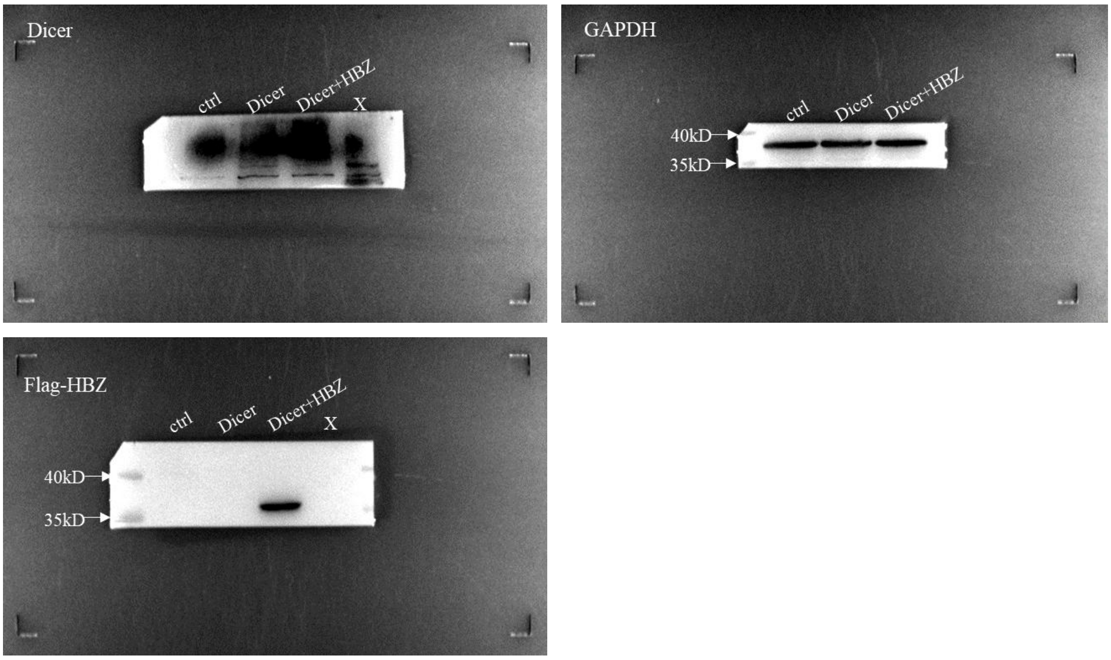

Figure 4D Western blot confirming the knockdown efficiency of Dicer in Jurkat-HBZ cells transduced with Dicer-specific shRNA (shDicer).

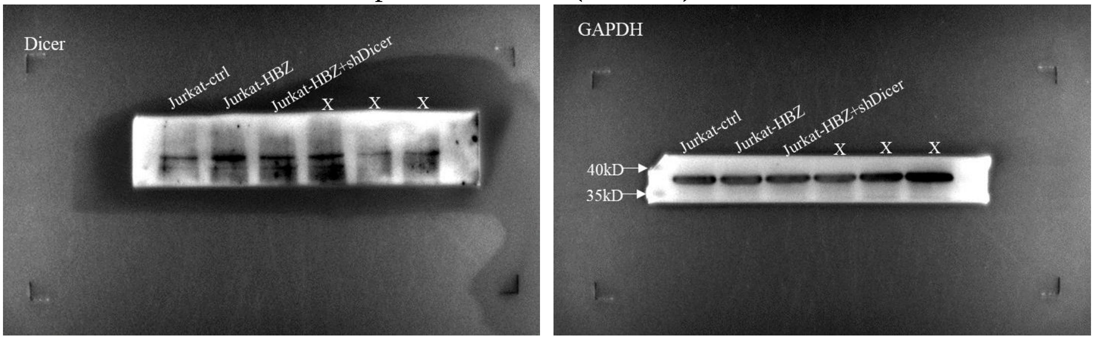

Figure 5D Western blot analysis of PTEN protein expression in rLV-miR-155 and rLV control cells.

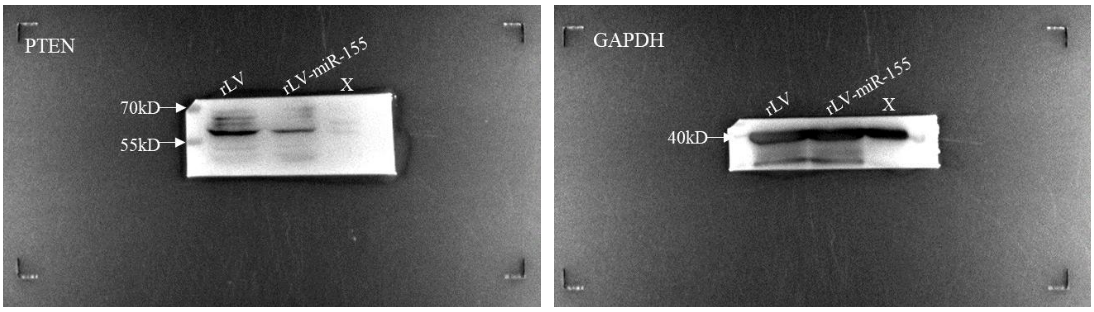

Figure 5E Western blot analysis of PTEN expression in ATL-T cells transfected with miR-155 inhibitor or negative control (NC).

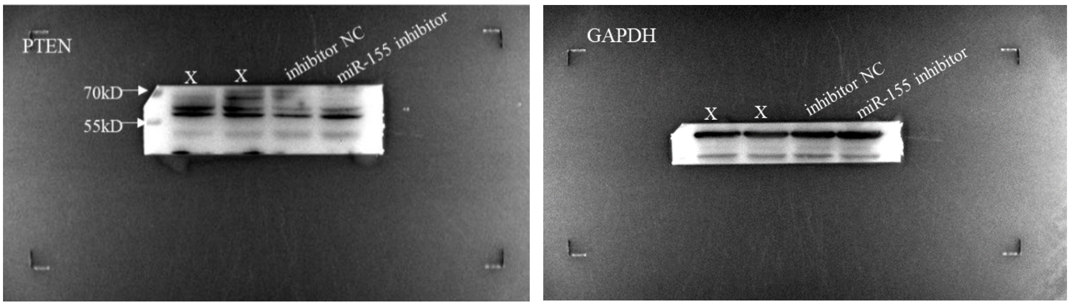

Figure 5F Western blot analysis of PI3K, phosphorylated Akt (p-Akt), and total Akt in rLV-miR-155 and rLV control cells.

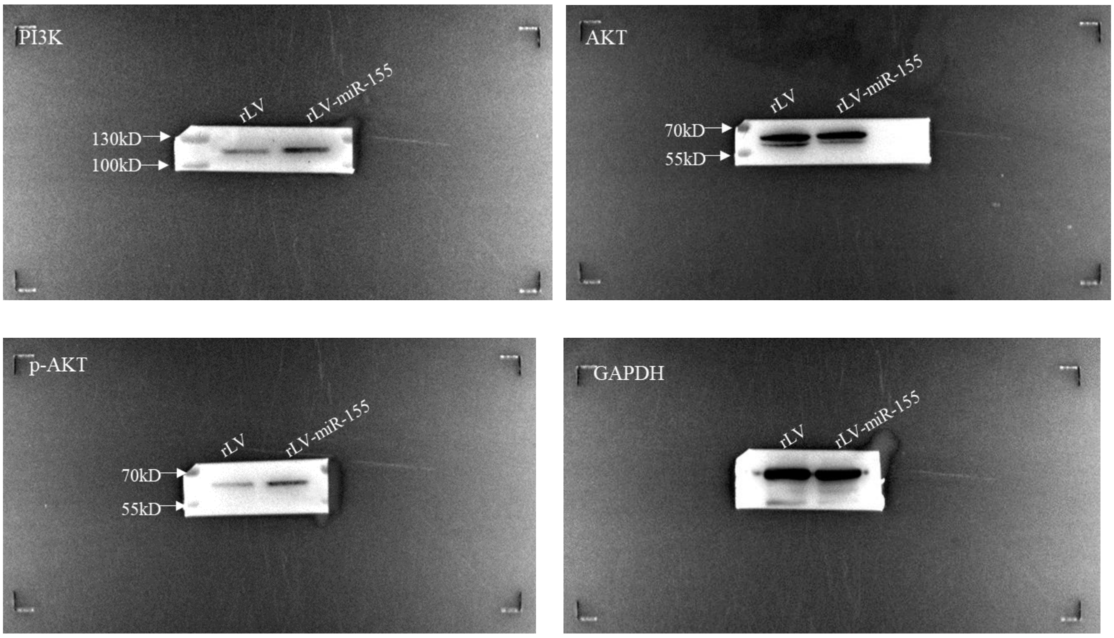

Figure 5G Western blot analysis of Tax, PTEN, PI3K, p-Akt, and total Akt in 293T cells co-transfected with pX1MT-T and siRNAs targeting Tax (siTax) or HBZ (siHBZ). Knockdown of either viral protein attenuated miR-155-mediated PTEN suppression and PI3K-Akt pathway activation.

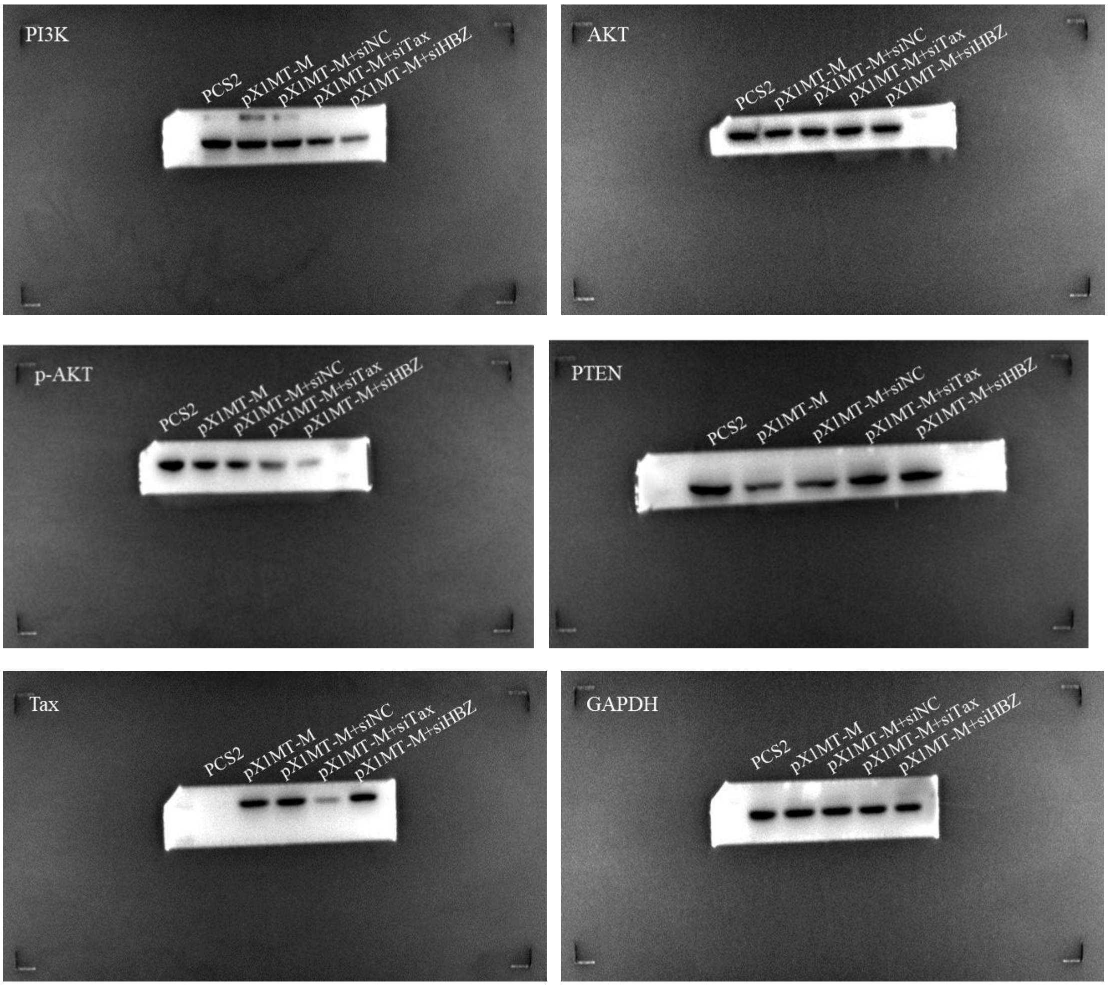

Figure 6F Western blot analysis of PTEN expression in rLV-miR-155 Jurkat cells transfected with PTEN expression vector (pCMV6-PTEN).

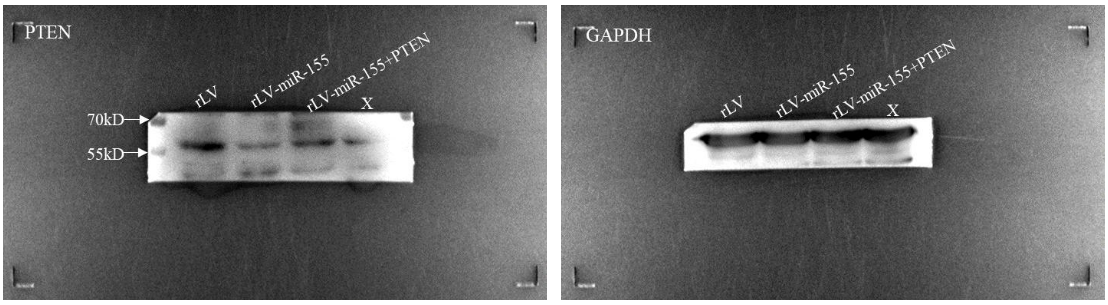

Figure S1A Western blot analysis of Tax expression in the JPX-9 cell line following treatment with 30 μmol/L $CdCl_2$ for the indicated durations (0, 6, 12 hours). GAPDH serves as a loading control.

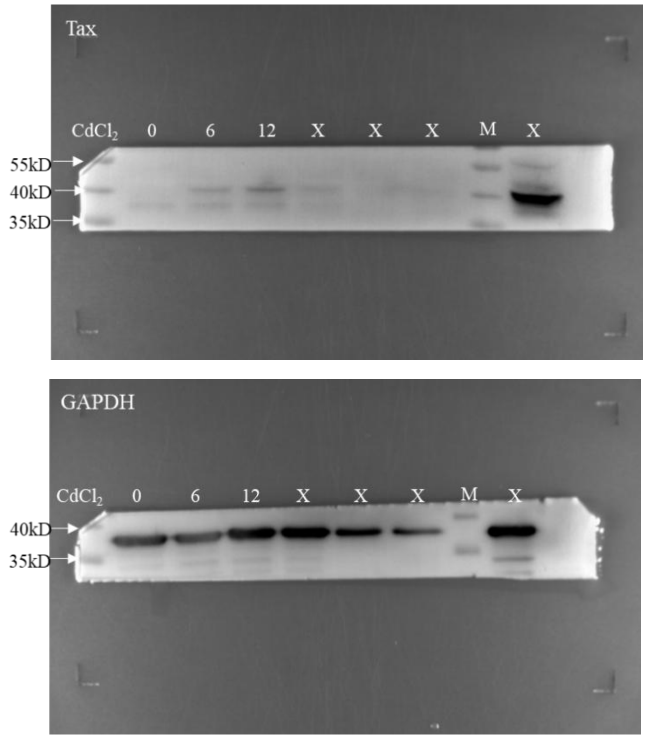

Figure S1F Knockdown of Tax or HBZ alone or together in HTLV-1-infected 293T cells.

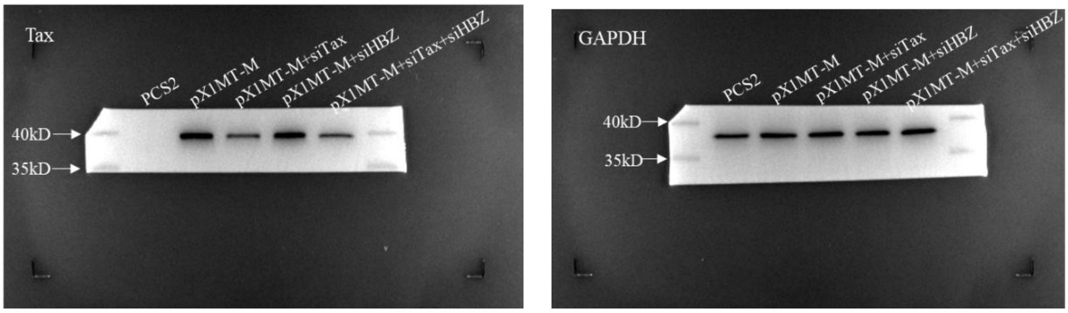

Figure S2D Co-immunoprecipitation (Co-IP) assay in 293T cells co-transfected with Flag-HBZ and V5-Dicer. Cell lysates were immunoprecipitated with anti-Flag antibody, and the precipitated proteins were detected by Western blot using anti-Flag and anti-Dicer antibodies.

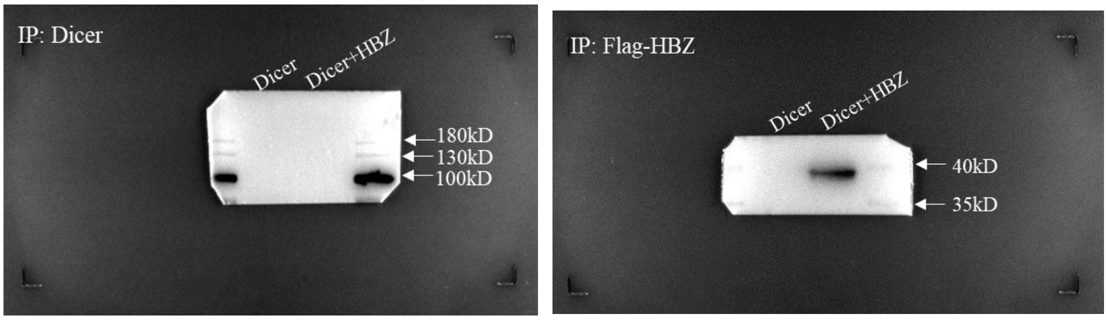

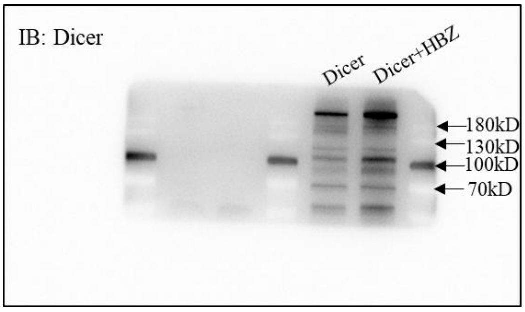
IB: Dicer
Dicer
Dicer+HBZ
180kD
130kD
100kD
70kD

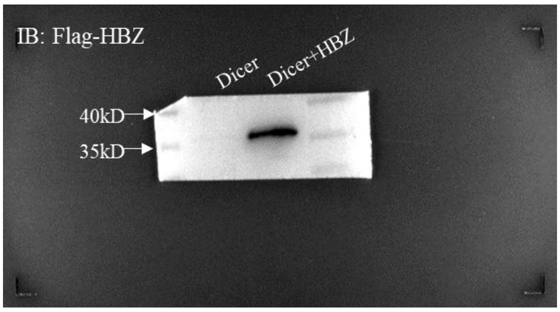
IB: Flag-HBZ
Dicer
Dicer+HBZ
40kD
35kD
