# Supplementary material for: HTLV-1 Tax and HBZ cooperatively promote leukemogenesis through miR-155-mediated PTEN suppression and PI3K-Akt activation
Source: J Virol. 2026 Jun 4;100(6):e00554-26. doi: 10.1128/jvi.00554-26 (PMC13288991; doi:10.1128/jvi.00554-26)
Supplement: Supplemental tables — Tables S1 and S2. [file jvi.00554-26-s0005.docx]

**Appendix** **Table 1. List of primers for real time PCR**

| **Target** | **Sequence** |
| --- | --- |
| miR-155 | TTAATGCTAATCGTGATAGGGG |
| pre-miR-155 | F: TTAATGCTAATCGTGATAGGGGTTT R: TGCTAATATGTAGGAGTCAGTTGG |
| HBZ | F: TGTTTCGATGCCTGCCTGTG R: GGATAATAGCCCGTCCACCA |
| Drosha | F: TCTCTGGAAAGGTCCTACAAAA R: CAGGTTCAGGAACAACCGATA |
| Dgcr8 | F: AAAACTTGCGAAGAATAAAGCT R: TCTGTTTAACAAAGTCAGGGATGA |
| Exportin-5 | F: TCAAAGGCGCAAGCAAACTG R: TCAGAATCTGCACCCAGCAAT |
| Dicer1 | F: GATGGTTCTCGAAGGCCCG R: AGCAACCTGGTTTGCAGAGT |
| Ago2 | F: GACACGAAAATCACCCACCC R: AGGACGTGATAGTGCGAAGG |
| Ago3 | F: GGGGCGTTCATTTTTCTCCG R: GGCAGGCCTCCTTGTTACAT |
| 18s | F: AACCCGTTGAACCCCATT R: CCATCCAATCGGTAGTAGCG |

**Appendix Table 2. Top 10 significantly up-regulated miRNAs in ATLL patients versus healthy controls**

This table lists the ten most significantly up‑regulated microRNAs identified through analysis of the GSE11577 dataset, selected based on the threshold of |LogFC| ≥ 1 and p‑value < 0.05. Each entry includes the miRNA identifier, corresponding log fold change (LogFC), and p‑value. miR‑155 is indicated as one of the most prominently up‑regulated miRNA.

| **miRNA** | **logFC** | **AveExpr** | **t** | **P.Value** |
| --- | --- | --- | --- | --- |
| hsa-miR-9* | 3.263492 | 9.087328 | 12.07054 | 3.26E-06 |
| hsa-miR-146a | 1.690376 | 14.83872 | 9.677818 | 1.58E-05 |
| **hsa-miR-155** | **2.378182** | **12.87989** | **6.785451** | **0.00018** |
| hsa-miR-9 | 1.872119 | 8.277513 | 6.432263 | 0.000256 |
| hsa-miR-7 | 2.591373 | 11.03992 | 5.388211 | 0.000788 |
| hsa-miR-96 | 1.107456 | 9.576033 | 5.329055 | 0.000844 |
| hsa-miR-182 | 1.804688 | 10.66185 | 5.120283 | 0.001076 |
| hsa-miR-141 | 1.220488 | 9.829373 | 4.80504 | 0.001573 |
| hsa-miR-106a | 0.958227 | 13.07057 | 4.795215 | 0.001592 |
| hsa-miR-153 | 2.224322 | 8.865946 | 4.461937 | 0.002415 |
